# A116 PROMOTING ALCOHOL CESSATION IN THE INPATIENT GASTROENTEROLOGY WARD

**DOI:** 10.1093/jcag/gwae059.116

**Published:** 2025-02-10

**Authors:** M Hussain, N Natt, Y Almahanna, D Hudson

**Affiliations:** Western University, London, ON, Canada; Gastroenterology, Western University, London, ON, Canada; Division of Gastroenterology, Department of Medicine, Western University Schulich School of Medicine & Dentistry, London, ON, Canada; Division of Gastroenterology, Department of Medicine, Western University Schulich School of Medicine & Dentistry, London, ON, Canada

## Abstract

**Background:**

Alcohol use disorder (AUD) is a significant global health issue, ranking as the third leading cause of death and disability, with a financial burden exceeding $16 billion annually for the Canadian healthcare system. A recent review of patient discharges from the inpatient Gastroenterology ward at University Hospital revealed that less than 8% of patients with AUD were discharged with anti-craving medications or addiction referrals, highlighting a gap in care coordination

**Aims:**

Increase the prescription rate of anti-craving medications by 20% for patients admitted with alcohol-related conditions to the Gastroenterology ward within the next 6 months, to improve post-discharge care and addiction support

**Methods:**

The project began by identifying key stakeholders and surveying 22 residents rotating through gastroenterology and 11 consultant physicians. Results revealed that 34.7% of residents and 36.4% of consultants were uncomfortable prescribing medications for alcohol use disorder. Root cause analysis identified several issues: limited knowledge of anticraving medications, lack of addiction resources, time constraints, and no standardized process for identifying high-risk alcohol use disorder patients.

Multiple Plan-Do-Study-Act (PDSA) cycles were implemented, targeting trainee education with handouts, early detection of high-risk patients using the AUDIT-C questionnaire at admission, and increasing referrals to addiction services and social work

**Results:**

Between January and July 2024, a total of 57 patients with alcohol-related admissions were identified. During the intervention period, educational handouts for trainees and a Gastroenterology educational grand rounds presentation were implemented, demonstrating some success in increasing rates of anticraving medications. However, these results were not sustained, highlighting the need for a long-term solution. We are developing an EMR-based admission order set to standardize AUDIT-C completion, facilitating the identification of high-risk inpatients and automatic consultations with social work and addiction services through informatics principles

**Conclusions:**

Although educational interventions temporarily increased rates of anticraving medications, the results were not sustainable. The project is ongoing and will transition to exploring EMR-based interventions

Comfort of Prescribing Anti-Craving Medication among GI staff

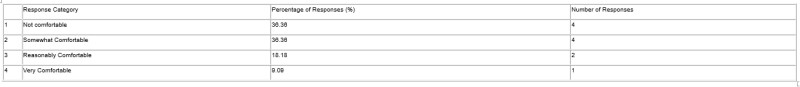

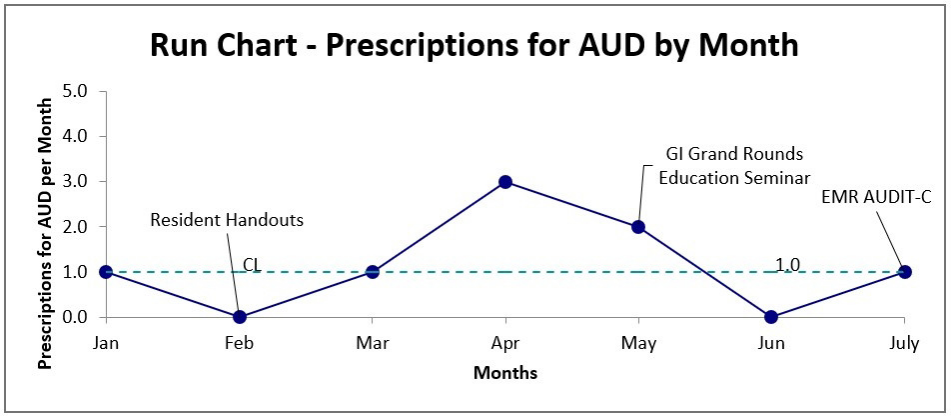

**Funding Agencies:**

**None**

